# Early-Life Air Pollution Exposure, Neighborhood Poverty, and Childhood Asthma in the United States, 1990–2014

**DOI:** 10.3390/ijerph15061114

**Published:** 2018-05-30

**Authors:** Nicole Kravitz-Wirtz, Samantha Teixeira, Anjum Hajat, Bongki Woo, Kyle Crowder, David Takeuchi

**Affiliations:** 1 Department of Emergency Medicine, University of California, Davis School of Medicine, Sacramento, CA 95817, USA; 2 School of Social Work, Boston College, Chestnut Hill, MA 02467, USA; samantha.teixeira@bc.edu (S.T.); woob@bc.edu (B.W.); takeuchi@bc.edu (D.T.); 3 Department of Epidemiology, School of Public Health, University of Washington, Seattle, WA 98195, USA; anjumh@uw.edu; 4 Department of Sociology, University of Washington, Seattle, WA 98195, USA; kylecrow@uw.edu

**Keywords:** asthma, children, prenatal, postnatal, early-life, air pollution, nitrogen dioxide, particulate matter, neighborhood poverty

## Abstract

Ambient air pollution is a well-known risk factor of various asthma-related outcomes, however, past research has often focused on acute exacerbations rather than asthma development. This study draws on a population-based, multigenerational panel dataset from the United States to assess the association of childhood asthma risk with census block-level, annual-average air pollution exposure measured during the prenatal and early postnatal periods, as well as effect modification by neighborhood poverty. Findings suggest that early-life exposures to nitrogen dioxide (NO_2_), a marker of traffic-related pollution, and fine particulate matter (PM_2.5_), a mixture of industrial and other pollutants, are positively associated with subsequent childhood asthma diagnosis (OR = 1.25, 95% CI = 1.10–1.41 and OR = 1.25, 95% CI = 1.06–1.46, respectively, per interquartile range (IQR) increase in each pollutant (NO_2_ IQR = 8.51 ppb and PM_2.5_ IQR = 4.43 µ/m^3^)). These effects are modified by early-life neighborhood poverty exposure, with no or weaker effects in moderate- and low- (versus high-) poverty areas. This work underscores the importance of a holistic, developmental approach to elucidating the interplay of social and environmental contexts that may create conditions for racial-ethnic and socioeconomic disparities in childhood asthma risk.

## 1. Introduction

Asthma is one of the most common chronic diseases among children in the United States (US), affecting an estimated 6.2 million American young people (8.4%) in 2015, half of whom suffered from an attack or episode due to uncontrolled asthma [1,2]. The resulting physical, social, and economic consequences for both children and families can be far-reaching [3,4]. According to the most recent available data, asthma is the third leading cause of hospitalization and, in 2014, accounted for approximately 820,000 emergency department visits among children under 15 years of age [5]. The annual direct health care cost of asthma has been estimated at $50.1 billion [6]. An additional $5.9 billion has been attributed to indirect costs, such as school absenteeism [6]. In 2013, for example, asthma accounted for 13.8 million lost school days among six to 17 year-olds [1].

It is well-documented that modifiable environmental conditions, including air pollution, are associated with various asthma-related outcomes [7,8,9,10]. Until recently, much of the research in this area has focused on acute exacerbations and other short-term consequences among individuals with existing asthma, including studies linking ambient air pollution exposure to increased emergency hospitalizations [11,12,13], reduced lung function [14,15,16,17], poor asthma control [18], and reduced response to asthma rescue medications [19]. Relatively less is known about the extent to which environmental conditions generally, and ambient air pollution more specifically, contribute to the development rather than exacerbation of asthma. While this question has received increased attention by researchers in more recent years [20], several unanswered questions remain.

In particular, those studies that have assessed the association of prior exposure with future asthma diagnosis often utilize samples from Europe and, to a lesser extent, China [21,22,23,24]. While this work provides important insights and opportunities for cross-national comparison, whether these findings are generalizable to the US context is less clear, as the US generally has lower levels of air pollution than Europe and China. Further, exposure to air pollution in the US, along with other social and environmental stressors, is distinctly patterned by race and space [25,26,27,28]. Recent research has begun to address these gaps, examining pollution exposure and subsequent asthma diagnosis in several cities across the US [29]; however, the geographic scope of these studies remains limited, often focused on just one or only a small number of American cities.

A recent area of growth in the research on asthma risk has been the exploration of the specific impact of air pollution exposure during gestation and in early life [29,30,31,32]. Several pathophysiological processes inform this body of work. In humans, considerable respiratory airway development occurs during the second and third trimesters of pregnancy and continues until three years of age [33,34,35]. It has been hypothesized, therefore, that exposure to environmental toxins, including air pollution, during these early stages of airway development and of rapid growth may be particularly consequential, as the respiratory epithelium of the developing lung may have increased permeability to toxins during this time [36]. Moreover, exposure to air pollution in the prenatal and early postnatal periods may induce epigenetic changes which are relevant to the development of asthma. For instance, several candidate gene studies have reported an association between prenatal and postnatal air pollution exposure and differential methylation, a key epigenetic mechanism, in genes involved in oxidative stress [37,38] and chronic inflammation [39,40,41,42].

At least three recent studies have documented associations of early life air pollution exposure with subsequent childhood asthma risk in the US. Nishimura and colleagues linked higher exposure to NO_2_ in the first year of life to subsequent asthma diagnoses in African American and Latino/a children across five geographic regions in the US and Puerto Rico [29]. Hsu and colleagues used a birth cohort from Boston, Massachusetts to show a relationship between PM_2.5_ exposure at 16 to 25 weeks gestation and increased risk of asthma onset by age six [32]. Similarly, Pennington and colleagues found that air pollution exposure during pregnancy and in the first year of life are associated with a higher risk of asthma development by age five among a birth cohort of children in Georgia [43]. However, given the relatively limited geographic scope of these studies, there remains a need for more research to clarify the impacts of environmental exposures, particularly air pollution exposure, during the prenatal period and shortly afterward, on future childhood asthma risk [44].

The present study builds on and addresses limitations in this literature by examining asthma diagnosis in childhood as a function of exposure to air pollution during the prenatal and early postnatal periods using a population-based, multilevel, longitudinal study of US children and their families. Given the tendency for social and environmental stressors to cluster together, this study also investigates the interaction between early-life exposures to air pollution and neighborhood poverty on subsequent childhood asthma risk. Children who are exposed to under-resourced neighborhoods during early life, including in utero, are more likely than children from higher-resource neighborhoods to face inadequate healthcare access, poorer nutrition, and higher levels of psychological stress that often accompany concentrated poverty, which may increase exposure to air pollution, as well as susceptibility to its adverse consequences [45,46,47]. To our knowledge, this is one of only a few studies of the effects of early-life exposure to air pollution and other contextual stressors, including exposure in utero and shortly thereafter, on subsequent asthma risk among a sample of children from across the US.

## 2. Materials and Methods

Household-level data are drawn from the 1990 to 2013 waves of the Panel Study of Income Dynamics (PSID) main interview, a longitudinal, replenishing study of US households which began in 1968 with a national sample of over 18,000 individuals and 5000 families. A wide array of information on demographic characteristics, socioeconomic position, health, geographic location, and related topics has been collected on adult household heads and their descendants annually through 1997 and biennially thereafter (i.e., 1999, 2001, 2003, 2005, 2007, 2009, 2011 and 2013). To gather more detailed information on children in PSID households, we use the Child Development Supplement (CDS), a research component of the PSID consisting of two cohorts. The first cohort included up to two children per household who were no more than 12 years old in 1997, and who were followed up in 2002/03 and 2007/08. The second cohort was initiated in 2014 and included all eligible children in PSID households born since 1997, and who are being followed through early adulthood. We hereafter refer to these as the 1997 and 2014 CDS cohorts, respectively. To be eligible for the 1997 or 2014 CDS cohorts, a child’s household must have responded to the 1997 or 2013 waves, respectively, of the PSID main interview. This study was approved by the University of Washington’s Institutional Review Board (Human Subjects Division Number: 43931).

### 2.1. Sample

Our sample comprises 5897 CDS respondents who were born between 1990 and 2011, years that correspond with available data on air pollution exposure, and for whom information on childhood asthma status was ascertained in the first CDS wave: 1997 for the 1997 CDS cohort and 2014 for the 2014 CDS cohort. We excluded 51 children for whom no parental identifiers exist, 59 children who would have been less than one year of age when asthma status was collected, as asthma diagnosis in infants is particularly difficult, 677 children with missing data on air pollution exposure in their year of birth and 575 children with missing data on one or more of our covariates. Our final analytic sample included 4535 children.

### 2.2. Exposure

Our primary exposure is ambient air pollution; specifically, annual-average concentrations of NO_2_ and PM_2.5_ in mothers’ census blocks of residence during their child(ren)’s year of birth (irrespective of birth month and day). As such, our exposure measures reflect a variable combination of pollutant exposure occurring both in utero and in the early postnatal period, depending on when during the year a child was born. When data on maternal pollution exposure were missing (*N* = 377), exposure data in fathers’ census blocks of residence were used as proxies. Given the relatively small geographic size of a census block, we consider these metrics as a proxy for individual-level exposure. NO_2_ is measured in parts per billion (ppb) and PM_2.5_ is measured in micrograms per cubic meter (µ/m^3^).

Data on NO_2_ for the period 1990 to 2011 are derived from the Environmental Protection Agency’s (EPA) Air Quality System (AQS), a database with ambient air pollution measurements collected from a nationwide network of monitoring stations. For PM_2.5_, AQS data from 1999 to 2011 are used for prediction. Predictions from 1990 to 1998 rely on a spatiotemporal modeling framework that extrapolated temporal trends back to 1990 [48]. Further, since air quality monitoring stations are unevenly distributed across regions of the country and vary over time, a combination of land-use regression (LUR), a multiple linear regression method based on geographic covariates, and universal kriging, which takes account of spatial dependence and spatial trends in the data, was used to spatially interpolate reliable annual-average air pollutant concentrations at the centroid of mothers’ (or fathers’) residential census blocks, the smallest unit of geography available in the PSID, for the 22 year period (1990–2011) during which sample children were born. This approach is described in more detail elsewhere [49,50] and has been applied in several recent epidemiologic studies of air pollution and health [51,52,53,54,55].

An additional exposure of interest is the poverty rate in mothers’ (or, if missing, fathers’) census tracts of residence in their child(ren)’s year of birth. We use a tract rather than a block-level measure for purposes of comparability with existing research on neighborhood poverty and health, and because many of the mechanisms through which such poverty-health effects are posited to operate do so at levels of geographic resolution beyond just the block [56]. Moreover, prior research suggests that measures of socioeconomic status at the census block and census tract (but not zip code) levels perform similarly across a number of different health outcomes [56]. Data on neighborhood poverty come from GeoLytics in which tract boundaries, which can change over time, have been normalized to 2010 definitions. We define low-, moderate-, and high-poverty neighborhoods, respectively, as those with a poverty rate less than 10%, poverty rates between 10% and 20%, and poverty rates greater than 20% [57].

### 2.3. Outcome 

Childhood asthma diagnosis is a self-reported measure based on a primary caregiver’s affirmative response to the CDS question, *“Has your doctor or health professional ever said that (CHILD) had asthma?”*, in either 1997 or 2014, depending on CDS cohort.

### 2.4. Covariates 

We consider a number of sociodemographic covariates which have shown prior associations with asthma [3,29,32]. Covariates at the child-level include: age, sex, CDS cohort, and whether either parent had a history of asthma. Household-level covariates include: age, sex, race, and employment status of the household head, smoking status of the household head and partner (“spouse”), household income (adjusted to year 2000 dollars) and, as a general indicator of housing quality, the number of persons per room in the household. Child-level covariates are drawn from the first CDS wave in 1997 or 2014, depending on CDS cohort. Household-level covariates come from the corresponding wave of the PSID main interview: 1997 for the 1997 CDS cohort and 2013 for the 2014 CDS cohort.

### 2.5. Statistical Analysis 

We use logistic regression and estimate odds ratios (OR) and 95% confidence intervals (CI) to examine the association of exposure to each neighborhood pollutant in children’s year of birth with future doctor-diagnosed asthma. To account for the non-independence of observations within families (e.g., siblings or children living in the same household at the time of asthma ascertainment), we estimate robust standard errors to account for clustering at the household level. Initial analyses examine the bivariate association of early-life pollution exposure with childhood asthma. Second, we use multiple logistic regression to evaluate these effects after adjustment first for sociodemographic covariates and second, for health-related covariates (i.e., household smoking, health insurance, and parental asthma history), in addition to the sociodemographic variables. Thirdly, we stratify the data by neighborhood poverty level and fit the fully adjusted models separately for respondents whose mothers were exposed to low-, moderate-, or high-poverty areas during their child(ren)’s year of birth. We examined models in which pollution values had been rescaled to represent either a one standard deviation (SD) increase (i.e., 6.59 ppb in NO_2_ and 3.31 µ/m^3^ in PM_2.5_), so as to make observed associations comparable across pollutants, or a fixed increase defined by the interquartile range (IQR) (i.e., 8.51 ppb in NO_2_ and 4.43 µ/m^3^ in PM_2.5_), so as to facilitate comparison with other studies. Additional sensitivity analyses include child low birthweight as a covariate, exclude non-singleton births (i.e., twins and triplets), and restrict the minimum age of children at the time of asthma ascertainment, as diagnoses before ages six to eight are more likely to be transient. 

## 3. Results

Table 1 presents summary statistics overall and by CDS cohort for our exposure and outcome variables, along with sociodemographic and health-related covariates. Of our 4535 respondents, 667 (14.71%) reported (via their primary caregiver) doctor-diagnosed asthma at the first CDS wave when they were, on average, about 7 years of age. Children’s average exposure [mean +/− standard deviation (SD)] to NO_2_ and PM_2.5_ in early life was 12.80 +/− 6.59 ppb and 12.73 +/− 3.31 µ/m^3^, respectively. For reference, the EPA’s annual National Ambient Air Quality Standard is 53 ppb for NO_2_ and 12 µ/m^3^ for PM_2.5_. Notably, however, existing research has increasingly found health risks associated with levels of NO_2_ well below the EPA annual standard, suggesting that this standard may be insufficient to protect children’s health [29]. Approximately 37% of respondents resided in low poverty neighborhoods in early life, while about 31% and 32% resided in moderate- and high-poverty neighborhoods, respectively. 

Consistent with prior research [29,32,43], our analyses revealed statistically significant associations between exposure to both NO_2_ and PM_2.5_ in early life and subsequent childhood asthma risk, both before and after adjustment for relevant covariates (Table 2 and Table 3). Because SD and IQR rescaling of the pollution variables produced substantively equivalent results, we present only the IQR-rescaled estimates throughout; though the SD-rescaled results are available in the supplemental material (supplemental Tables S1–S3). In practice, the IQR-rescaled estimates compare a child who was exposed to the middle of the upper half of the distribution of a particular pollutant (the 75th percentile, Q3) to a child who was exposed to the middle of the lower half of the distribution (the 25th percentile, Q1) or, in other words, a child exposed to a typical “high” level of pollution with a child exposed to a typical “low” level.

In multivariate analyses, adjustment for sociodemographic and health-related covariates had relatively small effects on the association of early-life pollution exposure and asthma risk. Including sociodemographic covariates slightly increased the magnitude of the pollutant effect estimates, with sex of both the child and the household head, race of the household head, CDS cohort (likely due to children’s older age, on average, in the 2014 vs. 1997 cohorts), and the number of persons per room in the household playing the largest moderating roles (Model 2). The further addition of health-related covariates in the fully-adjusted models (Model 3), particularly parental history of asthma and health insurance coverage, slightly attenuated the magnitude of the pollutant effect estimates, suggesting that these variables may help to explain at least part of our focal relationship; however, the relationship between an interquartile increase in pollutant exposure and the odds of childhood asthma diagnosis remained statistically significant.

The IQR for NO_2_ exposure among our final sample of children was 8.51 ppb (i.e., IQR = Q3−Q1 = 16.42−7.91 = 8.51). In the fully-adjusted analysis, an interquartile increase in children’s NO_2_ exposure during the prenatal and early postnatal periods increased the odds of future childhood asthma by a factor of 1.25 (95% confidence interval (CI) = 1.10–1.41) (Table 2, Model 3). Thus, a sample child exposed to a typical “high” concentration of NO_2_ in early life had a 1.25 higher odds of developing asthma than a sample child exposed to a typical “low” concentration of NO_2_. Coincidently, an interquartile increase in early-life exposure to PM_2.5_ (IQR = 14.89−10.47 = 4.43 µ/m^3^) also increased the odds of future childhood asthma by a factor of 1.25 (95% CI = 1.06–1.46).

In fully-adjusted analyses stratified by neighborhood poverty exposure in early life, the association between early-life air pollution and subsequent childhood asthma risk was only statistically significant in the moderate- and high-poverty neighborhoods, but not the low-poverty areas for NO_2_ and only in the most impoverished areas for PM_2.5_ (Table 4). Specifically, early-life NO_2_ exposure was not significantly associated with increased risk of future asthma among children exposed to low poverty neighborhoods in early life, whereas an interquartile increase (8.51 ppb) in early-life NO_2_ exposure increased the odds of future asthma among children exposed to moderate- and high-poverty neighborhoods in early life by a factor of 1.26 (95% CI = 1.02–1.57) and 1.31 (95% CI = 1.08–1.60), respectively. Similarly, early-life exposure to PM_2.5_ was not associated with future asthma risk among children simultaneously exposed to either low- or moderate-poverty neighborhoods; however, the odds of future asthma development among a sample child exposed to a typical “high” versus a typical “low” concentration of PM_2.5_ in early life were 1.35 times (95% CI = 1.03–1.78) greater when the child was also exposed to a high-poverty neighborhood.

### Sensitivity Analyses

We performed sensitivity analyses to test the robustness of our fully-adjusted results: (1) to the inclusion of child low birthweight as a covariate, (2) to the exclusion of non-singleton births (i.e., twins and triplets), and (3) to differing restrictions on the minimum age of children at the time of asthma ascertainment (as a proxy for the stability of the diagnosis). When we included a dichotomous indicator of child low birthweight, the associated coefficient was not statistically significant and the resulting ORs for both pollutants were largely unchanged. Our findings were also robust to the exclusion of 65 sets of twins and 3 sets of triplets from the analyses. Finally, it has been suggested that asthma diagnosis before ages six to eight years of age may be unstable [58]. Although we cannot determine the exact age at which children were diagnosed based on available CDS data, analyses excluding children who were less than six and eight years of age, respectively, when asthma status was ascertained produced results that were substantively similar to the above, though the magnitude of the ORs for early life pollution exposure were greater (results not shown, but available upon request).

## 4. Discussion

There is a small but growing body of literature that explores the connection of prenatal and early-life exposure to air pollution with future asthma risk. Recent meta-analyses have found significant associations between exposure to PM_2.5_, NO_2_, and risk of asthma development in childhood, despite some heterogeneity across studies [59,60]. Still, however, other meta-analyses looking at longitudinal birth cohort studies do not find statistically significant associations between pollution exposure and asthma causation [61]. This work points to a number of ongoing challenges in the literature, including heterogeneity in both the definition and measurement of asthma and varying exposure assessment methods for air pollution [62], as well as to the need for more research examining not only the association of air pollution with acute exacerbations of existing asthma-related conditions, but also with risk for developing asthma.

This study aims to contribute to this body of literature by examining childhood asthma as a function of exposure to air pollution during the prenatal and early postnatal periods using spatially- and temporally-resolved air pollution measures and a population-based, multilevel, longitudinal study of US children and their families. Consistent with prior research that found associations between prior pollution exposure and future asthma risk [29,32,43], our analyses reveal that the odds of future asthma diagnosis for children exposed to a high concentration of NO_2_ in early life are 1.25 times greater than those for children exposed to a low concentration of NO_2_. We also find that an interquartile increase in early-life exposure to PM_2.5_ (4.43 µ/m^3^) increases the odds of future childhood asthma risk by a factor of 1.25. These findings lend support to the hypothesis that early life pollution exposures may be relevant for asthma development. 

This study also investigates the impact of interactions between early-life exposures to air pollution and neighborhood poverty on subsequent asthma risk in childhood. In the US, neighborhood resources and hazards, including exposure to air pollution, are unequally distributed and patterned by race and socioeconomic position [25,26,27,28]. In analyses stratified by neighborhood poverty exposure in early life, we find that the association between early-life air pollution exposure and subsequent childhood asthma risk is only statistically significant in moderate- and high-poverty, but not low-poverty neighborhoods for NO_2_ and only in the most impoverished areas for PM_2.5_. Residents of low socioeconomic status (SES) neighborhoods may be more vulnerable to the health burdens of pollution due to compromised health status and a lack of prenatal health care access [45], which may help to explain this association. Furthermore, residents in high-poverty neighborhoods may lack alternate locations for outdoor recreation, which may increase the likelihood that they are exposed to the disproportionately high levels of pollution which tend to be associated with low-SES neighborhoods.

Furthermore, maternal exposure to chronic stressors, which are more likely to be present in distressed communities, is also a relevant correlate of childhood asthma and may help to further explain the relationship among early-life exposure to pollution, neighborhood poverty, and subsequent asthma risk. Previous research highlights that the combination of place-based and individual-level psychosocial stressors throughout the life course (e.g., during the prenatal years or infancy) is linked to maternal and child health disparities [63]. Living in distressed neighborhood environments (e.g., neighborhood poverty, limited access to resources) not only is linked to pollutant source locations and pollution exposure, but also can be associated with the degree and magnitude of chronic individual stress, which in turn can alter allostatic load and maternal immune systems leading to adverse children’s health outcomes, including increased susceptibility to air pollution and other hazards [63]. Our findings provide support for a holistic approach to elucidating the interplay of social and environmental contexts that may create conditions for disparities in childhood asthma risk. 

This study should be interpreted in the context of several study limitations. First, we rely on retrospective self-reports of children’s asthma diagnosis. Second, asthma diagnosis in young children is difficult and may reflect transient wheezing rather than asthma, per se. While including younger children may introduce some outcome misclassification, sensitivity analyses excluding children who were less than six and eight years of age, respectively, had minimal impact on our findings. Third, though we were able to control for many important predictors of asthma, we were unable to include measures of genetic susceptibility and other correlates of air pollution exposure and asthma. In addition, air pollution was ascertained at the census block level. Although blocks are relatively small geographic areas, the lack of individual, address-based residential data may introduce some measurement error. Furthermore, there is imprecision in the time frame associated with our air pollution exposures. That is, a child born at the beginning of the year would be assigned to an exposure that occurred mostly after birth, while a child born at the end of the year would receive largely an in utero exposure. While these issues deserve additional investigation, it is reasonable to assume that this measurement error is essentially random and therefore does not bias our estimates. 

Despite these limitations, our study contributes to the literature by showing that the deleterious effects of air pollution derived from smaller scale, geographically-limited studies in the US, and larger studies in other countries, are for the most part generalizable to this larger, population-based US sample. We also add support to other cohort studies that point to the significance of early-life pollution exposure for later childhood asthma risk [21,22]. Overall, we find that exposure to traffic-related air pollution (NO_2_) and industrial and natural sources of air pollution (PM_2.5_) are associated with subsequent childhood asthma risk. We find that neighborhoods of residence provide significant contexts in terms of childhood asthma; neighborhood poverty appears to exacerbate the effect of air pollution on childhood asthma risk and lower-poverty contexts may exert a protective effect against these pollution effects, which is consistent with a recent study examining the synergistic effects of other contextual/familial stressors and pollution [64]. These findings point to the importance of continuing to develop the literature around social and environmental stressors and their interactions to better understand children’s asthma risk and address chronic health conditions like asthma. They also reinforce the importance of public health measures that are sensitive to protective and exacerbating neighborhood factors in assessing the health effects of pollution.

## 5. Conclusions

In order to better understand health and illness, we must incorporate physiological, environmental, and social factors into research [65]. Our research suggests that social and environmental disparities exert a cumulative effect, with early life exposures to pollution and distressed neighborhoods affecting later asthma risk. This points to important future directions for research, including a deeper examination of neighborhood distress-related factors on the development and risk of asthma, and the exacerbation of traditional asthma triggers. Given the coincidence of high concentrations of air pollution and neighborhood poverty in minority populated neighborhoods, these findings also highlight the importance of examining the interactive effects of pollution and neighborhood distress in shaping significant racial and ethnic variations in asthma and other health conditions. Finally, these findings of significant repercussions of pollution in the earliest stages of life point to the need for additional work on the reciprocal relationships between neighborhood characteristics and inter-neighborhood mobility that may shape differences in cumulative exposures to health risks across the life course.

## Figures and Tables

**Table 1:** Summary statistics, PSID-CDS, 1990–2014.

	Total	1997 CDS Cohort	2014 CDS Cohort
	(*N* = 4535)	(*N* = 1319)	(*N* = 3216)
**Child-Level**			
Childhood asthma, no. (%)			
No	3868 (85.29)	1180 (89.46)	2688 (83.58)
Yes	667 (14.71)	139 (10.54)	528 (16.42)
Early-life NO_2_ (ppb), mean (SD)	12.80 (6.59)	15.93 (7.38)	11.51 (5.76)
Early-life PM_2.5_ (µ/m^3^), mean (SD)	12.73 (3.31)	14.97 (3.03)	11.81 (2.97)
Early-life NH poverty, no. (%) *			
Low (<10%)	1677 (37.00)	515 (39.04)	1162 (36.15)
Moderate (10–20%)	1391 (30.69)	386 (29.26)	1005 (31.27)
High (>20%)	1465 (32.32)	418 (31.69)	1047 (32.58)
Age (years), mean (SD)	7.15 (4.17)	3.86 (1.98)	8.49 (4.09)
Sex, no. (%)			
Male	2282 (50.32)	670 (50.80)	1612 (50.12)
Female	2253 (49.68)	649 (49.20)	1604 (49.88)
Parental asthma history, no. (%)			
No	3735 (82.36)	1075 (81.50)	2660 (82.71)
Yes	800 (17.64)	244 (18.50)	556 (17.29)
**Household-Level**			
HH age (years), mean (SD)	37.82 (10.52)	34.62 (9.04)	39.13 (10.81)
HH sex, no. (%)			
Male	3209 (70.76)	968 (73.39)	2241 (69.68)
Female	1326 (29.24)	351 (26.61)	975 (30.32)
HH race, no. (%)			
NL White	2394 (52.79)	744 (56.41)	1650 (51.31)
NL Black	1815 (40.02)	546 (41.39)	1269 (39.46)
NL Asian	24 (0.53)	1 (0.08)	23 (0.72)
NL Other/Multi	23 (0.51)	5 (0.38)	18 (0.56)
Latino	279 (6.15)	23 (1.74)	256 (7.96)
HH employment, no. (%)			
Unemployed	665 (14.66)	177 (13.42)	488 (15.17)
Employed	3870 (85.34)	1142 (86.58)	2728 (84.83)
Income (year 2000 $), mean (SD)	53,137 (89177)	49,821 (45549)	54,497 (101776)
Persons per room, mean (SD)	0.81 (0.40)	0.74 (0.32)	0.84 (0.43)
Current household smoking, no. (%)			
No	3367 (74.24)	913 (69.22)	2454 (76.31)
Yes	1168 (25.76)	406 (30.78)	762 (23.69)
Health insurance, no. (%)			
Insured	4332 (95.52)	1249 (94.69)	3083 (95.86)
Uninsured	203 (4.48)	70 (5.31)	133 (4.14)

NH = Neighborhood; HH = Household head; NL = Non-Latino; * *N* = 4533 due to missing data on NH poverty status.

**Table 2:** Association of early-life NO_2_ exposure with childhood asthma risk, PSID–CDS, 1990–2014 (*N* = 4535).

	Model 1		Model 2		Model 3	
	NO_2_		NO_2_ + Demo		NO_2_ + Demo + Hlt	
	OR	95% CI	OR	95% CI	OR	95% CI
Early-life NO_2_ exposure	**1.23**	(1.11–1.37)	**1.28**	(1.14–1.45)	**1.25**	(1.10–1.41)
**Child-Level**						
Age (years)			1.02	(0.99–1.04)	1.02	(0.99–1.04)
Sex						
Male (ref)			1.00		1.00	
Female			**0.74**	(0.63–0.88)	**0.73**	(0.62–0.87)
Cohort						
1997 (ref)			1.00		1.00	
2014			**1.89**	(1.44–2.49)	**1.88**	(1.42–2.48)
Parental asthma history						
No (ref)					1.00	
Yes					**2.50**	(2.03–3.07)
**Household-Level**						
HH age (years)			0.99	(0.99–1.00)	1.00	(0.99–1.01)
HH sex						
Male (ref)			1.00		1.00	
Female			**1.33**	(1.04–1.70)	**1.33**	(1.03–1.70)
HH race						
NL White (ref)			1.00		1.00	
NL Black			**1.70**	(1.36–2.11)	**1.72**	(1.38–2.13)
NL Asian			1.15	(0.34–3.95)	1.29	(0.34–4.90)
NL Other/Multi			0.70	(0.17–2.86)	0.71	(0.21–2.40)
Latino			0.97	(0.62–1.53)	1.15	(0.73–1.81)
HH employment						
Unemployed (ref)			1.00		1.00	
Employed			0.91	(0.69–1.20)	0.99	(0.74–1.32)
Income (year 2000 $)			1.00	(1.00–1.00)	1.00	(1.00–1.00)
Persons per room			**0.76**	(0.58–0.99)	0.79	(0.60–1.04)
Current smoking						
No (ref)					1.00	
Yes					1.13	(0.91–1.39)
Health insurance						
Insured (ref)					1.00	
Uninsured					**0.47**	(0.27–0.81)

HH = Household head; NL = Non-Latino; Effect estimates are for the IQR increase in NO_2_ (8.51 ppb); estimates in bold are statistically significant at *p* < 0.05.

**Table 3:** Association of early-life PM_2.5_ exposure with childhood asthma risk, PSID-CDS, 1990–2014 (*N* = 4535).

	Model 1		Model 2		Model 3	
	NO_2_		NO_2_ + Demo		NO_2_ + Demo + Hlt	
	OR	95% CI	OR	95% CI	OR	95% CI
Early-life PM_2.5_ exposure	**1.21**	(1.09–1.35)	**1.28**	(1.09–1.49)	**1.25**	(1.06–1.46)
**Child-Level**						
Age (years)			1.01	(0.99–1.04)	1.01	(0.98–1.04)
Sex						
Male (ref)			1.00		1.00	
Female			**0.75**	(0.63–0.88)	**0.74**	(0.62–0.88)
Cohort						
1997 (ref)			1.00		1.00	
2014			**1.99**	(1.46–2.71)	**1.97**	(1.44–2.69)
Parental asthma history						
No (ref)					1.00	
Yes					**2.53**	(2.06–3.10)
**Household-Level**						
HH age (years)			1.00	(0.98–1.01)	1.00	(0.99–1.01)
HH sex						
Male (ref)			1.00		1.00	
Female			**1.32**	(1.03–1.69)	**1.31**	(1.02–1.69)
HH race						
NL White (ref)			1.00		1.00	
NL Black			**1.65**	(1.32–2.06)	**1.68**	(1.34–2.09)
NL Asian			1.24	(0.33–4.62)	1.37	(0.34–5.55)
NL Other/Multi			0.75	(0.18–3.15)	0.77	(0.22–2.67)
Latino			1.01	(0.64–1.59)	1.19	(0.76–1.87)
HH employment						
Unemployed (ref)			1.00		1.00	
Employed			0.90	(0.69–1.19)	0.99	(0.74–1.31)
Income (year 2000 $)			1.00	(1.00–1.00)	1.00	(1.00–1.00)
Persons per room			0.77	(0.59–1.00)	0.80	(0.61–1.05)
Current smoking						
No (ref)					1.00	
Yes					1.11	(0.90–1.37)
Health insurance						
Insured (ref)					1.00	
Uninsured					**0.47**	(0.27–0.82)

HH = Household head; NL = Non-Latino; Effect estimates are for the IQR increase in PM_2.5_ (4.43 µ/m^3^); estimates in bold are statistically significant at *p* < 0.05.

**Table 4:** Association of early-life pollution exposure with childhood asthma risk by neighborhood (NH) poverty, PSID-CDS, 1990–2014.

	<10% NH Poverty		10%–20% NH Poverty		>20% NH Poverty	
	(*N* = 1634)		(*N* = 1391)		(*N* = 1465)	
	OR	95% CI	OR	95% CI	OR	95% CI
Early-life NO_2_ exposure	1.20	(0.93–1.58)	**1.26**	(1.02–1.57)	**1.31**	(1.08–1.60)
Early-life PM_2.5_ exposure	1.20	(0.90–1.61)	1.24	(0.92–1.68)	**1.35**	(1.03–1.78)

NH = Neighborhood; Effect estimates are for the IQR increase in each pollutant (8.51 ppb for NO_2_ and 4.43 µ/m^3^ for PM_2.5_); estimates in bold are statistically significant at *p* < 0.05; models are adjusted for child age and sex, CDS cohort, whether either parent had a history of asthma, the age, sex, race, and employment status of the household head, smoking status of the household head and their partner, household income, and household crowding.

## Supplementary Materials

The following are available online at http://www.mdpi.com/1660-4601/15/6/1114/s1, Table S1: SD-rescaled effects of early-life NO_2_ exposure on childhood asthma, PSID-CDS, 1990–2014, Table S2: SD-rescaled effects of early-life PM_2.5_ exposure on childhood asthma, PSID-CDS, 1990–2014, Table S3: SD-rescaled effects of early-life pollution exposure on childhood asthma by neighborhood poverty, PSID-CDS, 1990–2014.
