# Supplementary material for: Early-Life Air Pollution Exposure, Neighborhood Poverty, and Childhood Asthma in the United States, 1990–2014
Source: Int J Environ Res Public Health. 2018 May 30;15(6):1114. doi: 10.3390/ijerph15061114 (PMC6025399; doi:10.3390/ijerph15061114)
Supplement: Supplementary file 1 [file ijerph-15-01114-s001.pdf]

International Journal of
Environmental Research and Public Health

MDPI

Supplementary Materials

Table S1.

Table S1. Association of early-life NO_2 exposure* with childhood asthma risk, PSID-CDS, 1990-2014 (n=4535)

	Model 1		Model 2		Model 3	
	NO2		NO2 + Demo		NO2 + Demo + Hlt	
	OR	95% CI	OR	95% CI	OR	95% CI
Early-life NO_2 exposure	**1.18**	(1.09-1.27)	**1.21**	(1.20-1.33)	**1.19**	(1.08-1.31)
Child-level:						
Age (years)			1.02	(0.99-1.04)	1.02	(0.99-1.04)
Sex						
Male (ref)			1.00		1.00	
Female			**0.74**	(0.63-0.88)	**0.73**	(0.62-0.87)
Cohort						
1997 (ref)			1.00		1.00	
2014			**1.89**	(1.44-2.49)	**1.88**	(1.42-2.48)
Parental asthma history						
No (ref)					1.00	
Yes					**2.50**	(2.03-3.07)
Household-level:						
HH age (years)			0.99	(0.99-1.00)	1.00	(0.99-1.01)
HH sex						
Male (ref)			1.00		1.00	
Female			**1.33**	(1.04-1.70)	**1.33**	(1.03-1.70)
HH race						
NL White (ref)			1.00		1.00	
NL Black			**1.70**	(1.36-2.11)	**1.72**	(1.38-2.13)
NL Asian			1.15	(0.34-3.95)	1.29	(0.34-4.90)
NL Other/Multi			0.70	(0.17-2.86)	0.71	(0.21-2.40)
Latino			0.97	(0.62-1.53)	1.15	(0.73-1.81)
HH employment						
Unemployed (ref)			1.00		1.00	
Employed			0.91	(0.69-1.20)	0.99	(0.74-1.32)
Income (year 2000 $)			1.00	(1.00-1.00)	1.00	(1.00-1.00)
Persons per room			**0.76**	(0.58-0.99)	0.79	(0.60-1.04)
Current smoking						
No (ref)					1.00	
Yes					1.13	(0.91-1.39)
Health insurance						
Insured (ref)					1.00	
Uninsured					**0.47**	(0.27-0.81)

HH = Household head

NL = Non-Latino

*Note: effect estimates are for a one standard deviation (SD) increase in NO_2 (6.59 ppb); estimates in bold are statistically significant at $p<0.05$

Table S2.

Table S2. Association of early-life $PM_{2.5}$ exposure* with childhood asthma risk, PSID-CDS, 1990-2014 (n=4535)

	Model 1		Model 2		Model 3	
	NO2		NO2 + Demo		NO2 + Demo + Hlt	
	OR	95% CI	OR	95% CI	OR	95% CI
Early-life $PM_{2.5}$ exposure	**1.15**	(1.06-1.25)	**1.20**	(1.06-1.35)	**1.18**	(1.05-1.33)
Child-level:						
Age (years)			1.01	(0.99-1.04)	1.01	(0.98-1.04)
Sex						
Male (ref)			1.00		1.00	
Female			**0.75**	(0.63-0.88)	**0.74**	(0.62-0.88)
Cohort						
1997 (ref)			1.00		1.00	
2014			**1.99**	(1.46-2.71)	**1.97**	(1.44-2.69)
Parental asthma history						
No (ref)					1.00	
Yes					**2.53**	(2.06-3.10)
Household-level:						
HH age (years)			1.00	(0.98-1.01)	1.00	(0.99-1.01)
HH sex						
Male (ref)			1.00		1.00	
Female			**1.32**	(1.03-1.69)	**1.31**	(1.02-1.69)
HH race						
NL White (ref)			1.00		1.00	
NL Black			**1.65**	(1.32-2.06)	**1.68**	(1.34-2.09)
NL Asian			1.24	(0.33-4.62)	1.37	(0.34-5.55)
NL Other/Multi			0.75	(0.18-3.15)	0.77	(0.22-2.67)
Latino			1.01	(0.64-1.59)	1.19	(0.76-1.87)
HH employment						
Unemployed (ref)			1.00		1.00	
Employed			0.90	(0.69-1.19)	0.99	(0.74-1.31)
Income (year 2000 $)			1.00	(1.00-1.00)	1.00	(1.00-1.00)
Persons per room			0.77	(0.59-1.00)	0.80	(0.61-1.05)
Current smoking						
No (ref)					1.00	
Yes					1.11	(0.90-1.37)
Health insurance						
Insured (ref)					1.00	
Uninsured					**0.47**	(0.27-0.82)

HH = Household head

NL = Non-Latino

*Note: effect estimates are for a one standard deviation (SD) increase in $PM_{2.5}$ (3.31 μ/m3); estimates in bold are statistically significant at $p<0.05$

Table S3.

Table S3. Association of early-life pollution exposure* with childhood asthma risk by neighborhood (NH) poverty, PSID-CDS, 1990-2014

	<10% NH Poverty (n=1634)		10%-20% NH Poverty (n=1391)		> 20% NH Poverty (n=1465)	
	OR	95% CI	OR	95% CI	OR	95% CI
Early-life NO_2 exposure	1.16	(0.94-1.42)	**1.20**	(1.02-1.42)	**1.24**	(1.06-1.44)
Early-life $PM_{2.5}$ exposure	1.15	(0.92-1.43)	1.18	(0.94-1.47)	**1.25**	(1.02-1.54)

*Note: effect estimates are for the standard deviation (SD) increase in each pollutant (6.59 ppb for NO_2 and 3.31 μ/m3 for $PM_{2.5}$); estimates in bold are statistically significant at $p<0.05$

© 2018 by the authors. Licensee MDPI, Basel, Switzerland. This article is an open access article distributed under the terms and conditions of the Creative Commons Attribution (CC BY) license (http://creativecommons.org/licenses/by/4.0/).
